# Geographical and temporal distribution of the residual clusters of human leptospirosis in China, 2005–2016

**DOI:** 10.1038/s41598-018-35074-3

**Published:** 2018-11-09

**Authors:** Pandji Wibawa Dhewantara, Abdullah Al Mamun, Wen-Yi Zhang, Wen-Wu Yin, Fan Ding, Danhuai Guo, Wenbiao Hu, Ricardo J. Soares Magalhães

**Affiliations:** 1 UQ Spatial Epidemiology Laboratory, School of Veterinary Science, The University of Queensland, Gatton, QLD 4343 Australia; 2 National Institute of Health Research and Development (NIHRD), Ministry of Health of Indonesia, Pangandaran Unit of Health Research and Development, Pangandaran, West Java, 46396 Indonesia; 3 Institute for Social Science Research, The University of Queensland, Indooroopilly, QLD 4068 Australia; 4 Center for Disease Surveillance and Research, Institute of Disease Control and Prevention of PLA, Beijing, 100071 People’s Republic of China; 5 Chinese Center for Disease Control and Prevention, Beijing, 102206 People’s Republic of China; 6 Computer Network Information Center, Chinese Academy of Sciences, Beijing, 100190 People’s Republic of China; 7 School of Public Health and Social Work, Queensland University of Technology, Kelvin Grove, QLD 4059 Australia; 8 Children’s Health and Environment Program, Child Health Research Centre, The University of Queensland, South Brisbane, QLD 4101 Australia

## Abstract

Human leptospirosis outbreaks still persistently occur in part of China, indicating that leptospirosis remains an important zoonotic disease in the country. Spatiotemporal pattern of the high-risk leptospirosis cluster and the key characteristics of high-risk areas for leptospirosis across the country are still poorly understood. Using spatial analytical approaches, we analyzed 8,158 human leptospirosis cases notified during 2005–2016 across China to explore the geographical distribution of leptospirosis hotspots and to characterize demographical, ecological and socioeconomic conditions of high-risk counties for leptospirosis in China. During the period studied, leptospirosis incidence was geographically clustered with the highest rate observed in the south of the Province of Yunnan. The degree of spatial clustering decreased over time suggesting changes in local risk factors. However, we detected residual high-risk counties for leptospirosis including counties in the southwest, central, and southeast China. High-risk counties differed from low-risk counties in terms of its demographical, ecological and socioeconomic characteristics. In high-risk clusters, leptospirosis was predominantly observed on younger population, more males and farmers. Additionally, high-risk counties are characterized by larger rural and less developed areas, had less livestock density and crops production, and located at higher elevation with higher level of precipitation compare to low-risk counties. In conclusion, leptospirosis distribution in China appears to be highly clustered to a discrete number of counties highlighting opportunities for elimination; hence, public health interventions should be effectively targeted to high-risk counties identified in this study.

## Introduction

Leptospirosis, an emerging yet neglected zoonotic disease caused by the pathogenic spirochetes belong to the genus *Leptospira*, has been a significant global public health hazard^1^. Infection can be asymptomatic or can manifest as a life-threatening disease due to acute renal failure, liver injury or pulmonary hemorrhage syndrome^2^. Annually worldwide, leptospirosis is estimated to cause more than one million cases, 58,900 deaths, and the loss of more than 2.90 million disability-adjusted life-years (DALYs)^3,4^. The high incidence occurs during wet seasons and flooding reaching to more than 100 per 100,000^5^. Human infection occurs via direct contact between injured skin or mucous membrane with the urine or blood containing the bacteria of the infected animals or due to exposure to bacterial-contaminated soil or water. At present, a total of 10 pathogenic *Leptospira* and 5 intermediate species have been identified so far and it is likely that novel species will be continuously discovered^6,7^. *Leptospira* could be carried by wide-range animals such as pigs, cattle and dogs, but rodents act as an eminent role in shedding the bacteria into the environment^8,9^. The spatial variation of leptospirosis incidence has been known to be driven by ecological (e.g., precipitation, elevation, animal hosts, land use types) and anthropogenic factors (e.g., farming activities, poverty)^10–14^.

In China, since the 1950s there were more than 2.5 million cases and approximately 20,000 deaths reported to the national disease notification system^15^. Within the last two decades, it was estimated that at least 10,000 disability-adjusted life-years (DALYs) lost because of leptospirosis and it was disproportionately affected males, young populations, and farmers^16^. *Leptospira interrogans* serogroup Icterohaemorrhagiae serovar Lai has been responsible for most human infections in China and *Apodemus agrarius* is the most important animal host among other animals such as pigs, cattle and dogs^17–19^. Leptospirosis cases have been notified in almost all provinces in China except the provinces of Ningxia and Xizang^16,20,21^. The geographical distribution of leptospirosis in China has been associated with climatic factors where the majority of incidence occur in tropical and sub-tropical regions in the southwest, central, south, and southeast of China^11,17,21^. A recent study suggested that physical environmental and socioeconomic characteristics could also play important role on preserving leptospirosis transmission in China^11^. However, further investigation is required to improve our understanding of the characteristics of high-risk areas of leptospirosis throughout the country. A better understanding of such characteristics would help guide health authorities at identifying potential areas for leptospirosis transmission as well as to target vulnerable population.

During the last two decades, there was a decline in the number of notified leptospirosis cases and mortality in China, which might be partly due to the effectiveness of control programmes deployed by Chinese authorities including rodent control, improvement in sanitation conditions, and vaccination during epidemic season especially in high-risk communities^22,23^. However, local leptospirosis outbreaks are still occurring in certain parts of the country^24–27^ indicating that leptospirosis remains an important zoonotic disease in the country. However, changes in the geographical distribution of leptospirosis incidence in China during the last decades, has not been adequately explored. More importantly, little is known about the location of residual high-risk foci of leptospirosis and key demographic, ecological and socio-economic characteristics that could explain residual disease transmission in those areas. This knowledge gap hinders the design and implementation of targeted interventions towards reducing risk and eliminating leptospirosis in China.

Geographic information systems (GIS)-based technologies have now been widely used in numerous infectious disease studies including in the field of leptospirosis^12,13,28^. It allows researchers and health authorities to better explore and understand the disease pattern and its underlying determinants. GIS can be used to map disease rates and help locate and characterize high-risk areas where interventions should be conducted. By combining GIS and spatial statistics, social and environmental risk factors associated with high-risk areas could be determined.

The aims of this study are (i) to investigate whether or not the spatial pattern of leptospirosis incidence was clustered over China during the study period, (ii) to identify the location of high- and low-risk counties for leptospirosis and (iii) to characterize high-risk counties by identifying differences between them and other type of counties in terms of their demographical, ecological and socioeconomic conditions. These research aims fit with the current gap in knowledge in terms of modifiable factors that distinguish high-risk form low risk areas that could be targeted for the design of local interventions. Findings from the present study would have much value for policymaking, especially at county-level, to strengthen disease surveillance programs and intervention strategies for leptospirosis.

## Results

### Descriptive analysis

**Figure 1:**
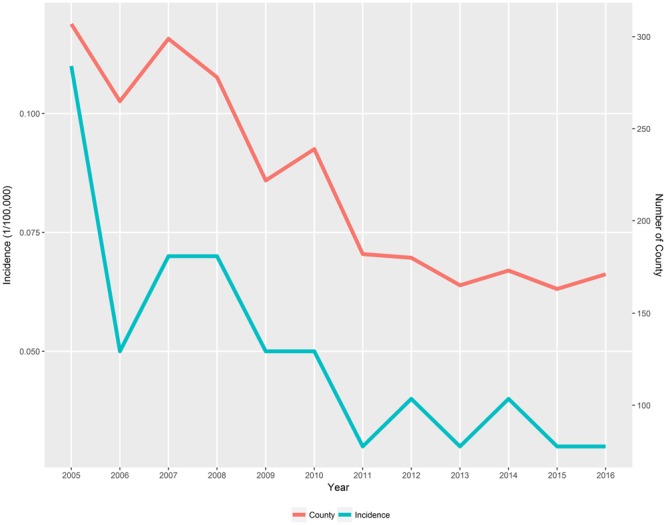
Annual notified incidence rate (per 100,000 people) and number of counties with human leptospirosis in China, 2005–2016. The graph was created by in R environment using ‘ggplot2’ package.

A total of 8,158 human leptospirosis cases were notified during 2005–2016 in 794 counties from total of 2,922 counties. Of which, 2,633 cases (32.27%) were laboratory confirmed cases. During 2005–2016, the notified incidence decreased as well as the number of counties with leptospirosis (Fig. 1). Incidence dropped after 2005, but there was a slight increase in rates during 2007–2008 before incidence continued to decrease until 2016. The number of counties with leptospirosis appears to have a similar pattern to that of the number of reported cases. The number of counties decreased over time but was relatively stable during 2011–2016 ranging from 163 to 182 counties (Supplementary Table A).

**Figure 2:**
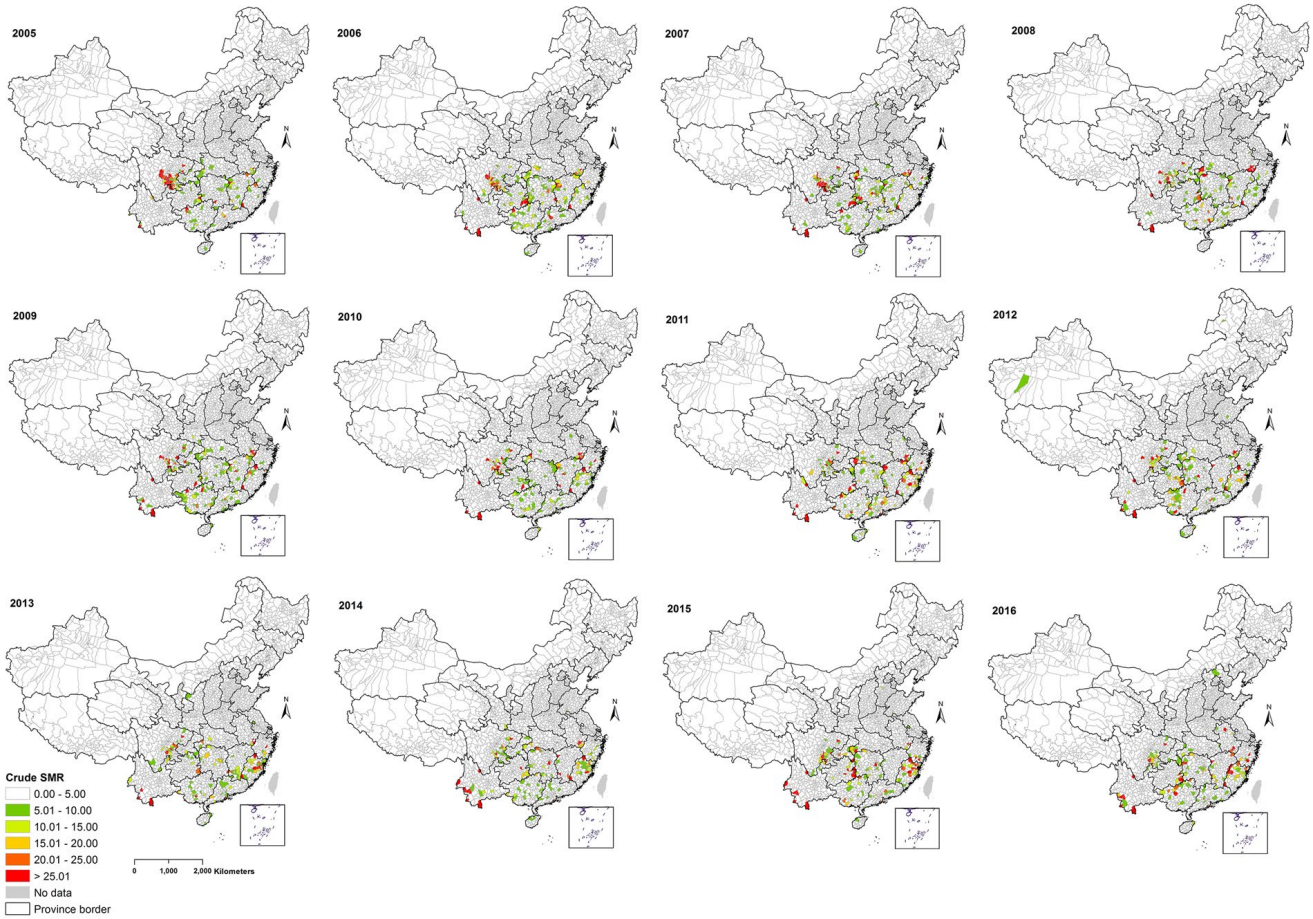
Crude standardized morbidity ratios (SMRs) for human leptospirosis by counties in China, 2005–2016. The map was created in ArcGIS 10.5.1 software, ESRI Inc., Redlands, CA, USA, (https://www.arcgis.com/features/index.html).

**Figure 3:**
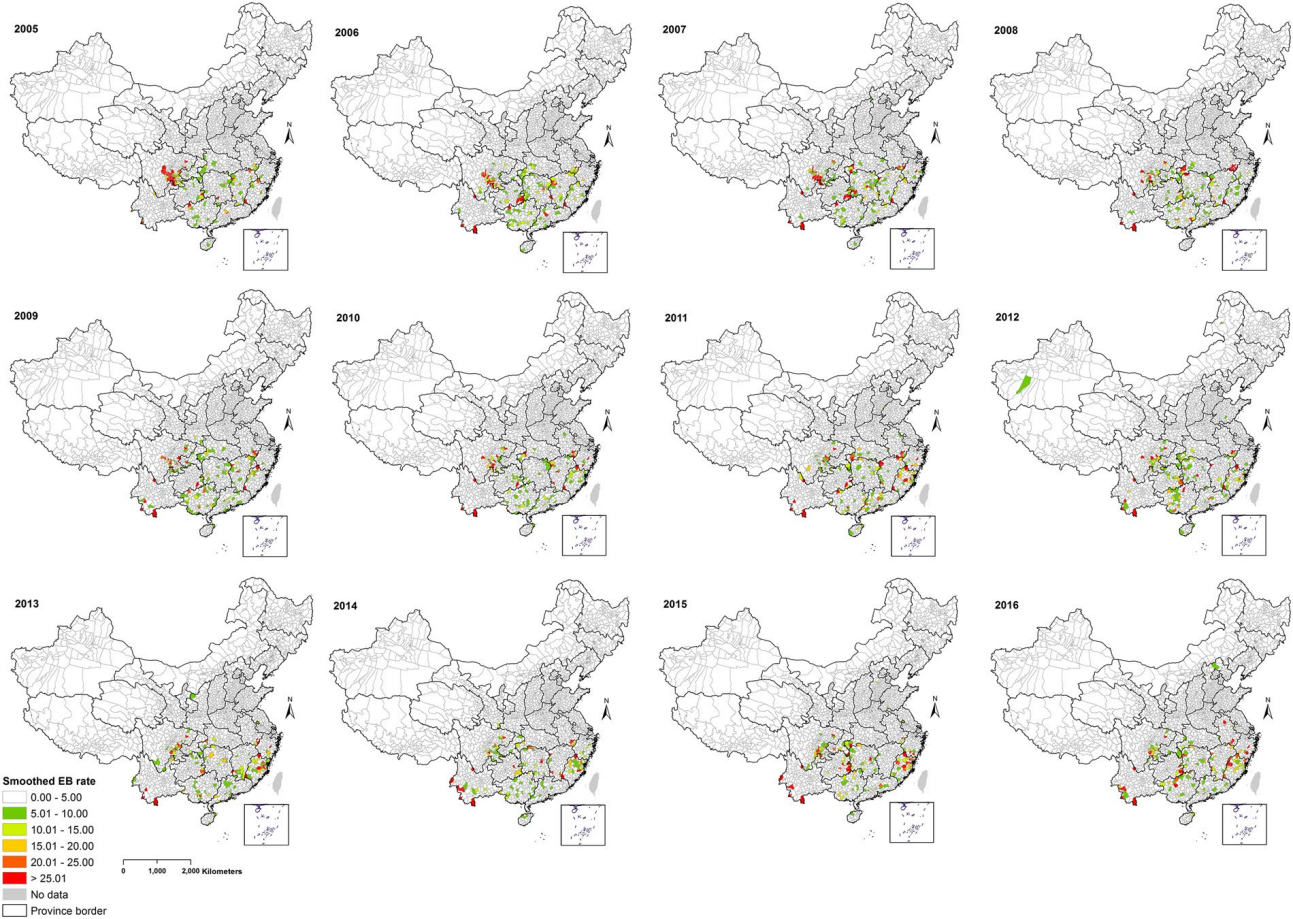
County-level smoothed rates maps of human leptospirosis using empirical Bayesian estimates, China, 2005–2016. The map was created in ArcGIS 10.5.1 software, ESRI Inc., Redlands, CA, USA, (https://www.arcgis.com/features/index.html).

Our results indicate geographical and temporal variation in the crude standardized morbidity ratios (SMRs) of notified human leptospirosis in China at county-level (Fig. 2). The smoothed SMRs maps reveal a clear distribution of counties with relatively high leptospirosis rates and also gradual changes in rates at the county level in China during 2005–2016 (Fig. 3). Two counties in the south of Yunnan province including Xishuangbanna Prefecture City (Mengla County) and Pu’er Prefecture City (Menglian County) consistently had the highest rate during 2005–2016. High smoothed rates were also observed in counties situated in the southeast of Sichuan, in the southeast Guizhou border to Hunan and Guangxi, north Fujian and southern Anhui.

### Spatial autocorrelation analysis

The Moran’s *I* analysis demonstrates a significant positive spatial autocorrelation in rates throughout the period studied, indicating that leptospirosis incidence was spatially clustered. Yet, there was a decreasing trend in the Moran’s value over time and reached the lowest value in 2013 (*I* = 0.009, *P*-value = 0.03) (Table 1).

**Table 1:** Spatial autocorrelation (Global Moran’s I) of human leptospirosis in China from 2005–2016.

Year	Moran’s I	*P*-value
2005	0.3167	0.001
2006	0.0390	0.011
2007	0.0711	0.004
2008	0.0841	0.001
2009	0.0404	0.013
2010	0.0308	0.011
2011	0.0376	0.003
2012	0.0373	0.016
2013	0.0102	0.032
2014	0.0097	0.033
2015	0.0232	0.012
2016	0.0198	0.015

**Figure 4:**
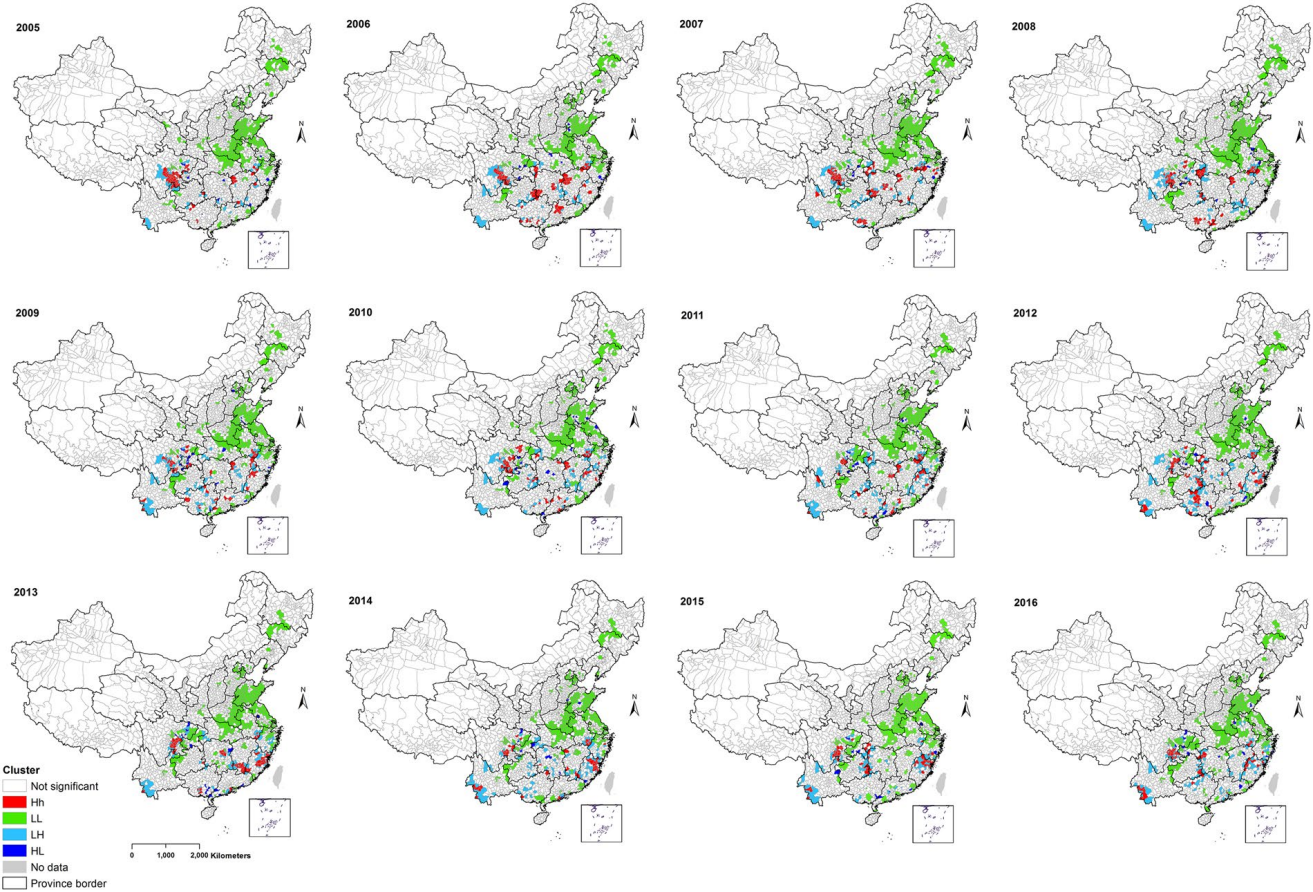
Annual spatial clusters pattern of human leptospirosis as determined by local indicator spatial autocorrelation (LISA), China, 2005–2016. The High-High (HH) (later stated as high-risk) cluster defined when they have high values surrounded by high values. Low-low (LL) (low-risk) clusters represented cluster of low rates surrounded low rates counties. Low-high (LH) or high-low (HL) was defined if a cluster of low or high rates values surrounded by high or low rates. The map was created in ArcGIS 10.5.1 software, ESRI Inc., Redlands, CA, USA, (https://www.arcgis.com/features/index.html).

Local indicator spatial association (LISA) test identified high-risk counties (classified HH clusters; red color) in southwestern provinces (e.g., Sichuan, Guizhou, Yunnan), central province (e.g., Hunan), southeastern provinces (e.g., Fujian, Anhui, Jiangxi, Zhejiang) and south China provinces (e.g., Guangxi and Guangdong) (Fig. 4). Low-risk counties (LL clusters; green color) were predominantly detected in provinces in the east towards northeast China.

The annual incidence rate in high-risk clusters fluctuated during the study period, ranging from 0.28 to 2.67 per 100,000 people with the highest rates observed in 2005. The number of high-risk counties was reduced 25% from 64 in 2005 to 48 counties in 2016 (Table 2). In total, there were 265 (10.35%) counties in 12 provinces classified as high-risk clusters during 2005–2016 (Table 3). A high proportion of high-risk counties relative to their total counties observed in Fujian (41%), Guangxi (32%), and Sichuan (31%). From 2005 to 2016, high-risk counties were consistently observed in the provinces of Yunnan, Sichuan, Guizhou, Fujian, and Anhui. In particular, four counties including Yanjin (Yunnan province), Yibin and Qianwei (Sichuan province), and Shexian (Anhui province) were high-risk counties for 10 years of the period studied.

**Table 2:** Descriptive statistics of human leptospirosis clusters, China, 2005–2016.

Year	Cluster^a^	No. of cases	Rates per 100,000	No. of counties	Type of the counties^b^		Population at risk
					Rural	Urban	
2005	H-H	757	2.67	64	62	2	28,477,361
	H-L	32	0.70	9	8	1	4,582,405
	L-H	12	0.07	45	45	0	17,408,650
2006	H-H	237	0.78	72	71	1	30,219,755
	H-L	9	0.09	11	11	0	9,878,203
	L-H	29	0.12	75	69	6	24,206,070
2007	H-H	290	1.04	64	60	4	27,915,298
	H-L	17	0.37	8	8	0	4,545,932
	L-H	68	0.32	64	59	5	21,000,362
2008	H-H	267	0.94	58	56	2	28,431,877
	H-L	18	0.34	9	9	0	5,263,666
	L-H	13	0.05	71	67	4	25,248,350
2009	H-H	113	0.48	53	51	2	23,175,690
	H-L	20	0.13	18	18	0	15,972,238
	L-H	68	0.19	89	83	6	35,524,615
2010	H-H	302	0.93	59	57	2	32,040,701
	H-L	11	0.07	17	16	1	14,364,705
	L-H	26	0.07	95	87	8	35,361,925
2011	H-H	110	0.48	45	37	8	22,927,200
	H-L	15	0.14	15	14	1	10,563,439
	L-H	29	0.07	107	96	11	41,261,003
2012	H-H	75	0.28	56	51	5	25,891,146
	H-L	1	0.01	10	10	0	8,416,354
	L-H	32	0.08	104	97	7	38,346,349
2013	H-H	133	0.40	60	52	8	32,470,676
	H-L	41	0.34	16	16	0	11,987,768
	L-H	33	0.09	86	79	7	38,621,772
2014	H-H	207	0.98	47	44	3	21,169,075
	H-L	3	0.04	11	10	1	7,821,347
	L-H	44	0.10	113	104	9	45,010,020
2015	H-H	86	0.49	45	45	0	17,341,826
	H-L	7	0.06	12	11	1	10,492,490
	L-H	66	0.19	98	89	9	33,872,308
2016	H-H	147	0.72	48	46	2	20,300,837
	H-L	23	0.16	16	14	2	14,518,982
	L-H	1	0.00	112	104	8	46,584,188

^a^H-H, high-high (high-risk); H-L, high-low; L-H, low-high. The High-High (HH) (later stated as high-risk) cluster defined when they have high values surrounded by high values. Low-low (LL) (low-risk) clusters represented cluster of low rates surrounded low rates counties. Low-high (LH) or high-low (HL) was defined if a cluster of low or high rates values surrounded by high or low rates.

^b^Type of counties defined based on the predominant proportion of area calculated from mean values of pixels of gridded raster urban-rural maps^51^.

**Table 3:** Yearly number of high-risk counties (n = 265) in each province as identified by local indicator spatial association (LISA), China, 2005–2016.

Province	Total counties (% of high-risk counties)	Number of high-risk counties												No. of cases (% total cases)
		2005	2006	2007	2008	2009	2010	2011	2012	2013	2014	2015	2016	
Guangdong	24 (20.2)	0	6	1	2	4	7	10	2	4	1	0	2	661 (8.10)
Guangxi	35 (31.8)	4	8	6	11	7	3	3	10	4	2	0	2	569 (6.97)
Zhejiang	7 (7.8)	0	0	4	1	0	1	0	0	0	1	2	1	149 (1.83)
Anhui	10 (9.5)	2	6	4	6	6	5	5	5	3	6	1	3	358 (4.39)
Fujian	35 (41.2)	1	2	0	0	5	5	7	7	18	14	14	7	535 (6.56)
Jiangxi	25 (25)	6	11	5	6	7	5	5	2	7	3	0	2	442 (5.42)
Hubei	7 (6.8)	0	2	4	6	0	1	1	2	0	0	2	0	299 (3.67)
Hunan	35 (28.7)	0	10	12	1	1	2	5	5	5	2	4	4	683 (8.37)
Chongqing	5 (13.2)	2	0	0	0	2	0	0	0	0	0	0	1	208 (2.55)
Sichuan	57 (31.1)	43	20	22	19	17	23	5	13	15	13	13	18	2410 (29.54)
Guizhou	11 (12.5)	1	4	2	3	0	3	1	6	1	0	5	2	297 (3.64)
Yunnan	14 (10.9)	5	3	4	3	4	4	3	4	3	5	4	6	1415 (17.34)

In general, the demographical, ecological and socioeconomic characteristics among clusters was significantly differed (p < 0.001) (Table 4). The characteristics of age, gender, and occupation was occupation statistically differ (p < 0.001) between clusters. Leptospirosis infections in high-risk clusters were observed in relatively younger groups (median 35; interquartile range, IQR: 21–47, p < 0.001) compared with cases reported in other types of clusters. In contrast, more leptospirosis cases were observed among older population in low-risk clusters (48, IQR: 34–57). Overall, the high number of leptospirosis case was observed in males than females (p < 0.001) in all clusters, but high-risk clusters had relatively higher proportion of case in males than that in low-risk cluster. Additionally, the high-risk clusters had more farmers (80.20%, p < 0.001) compared to other cluster types.

**Table 4:** Comparative analysis of demographic, ecological and socioeconomic variables stratified by four types of spatial clusters as determined by LISA, China, 2005–2016.

Characteristics	Cluster					F/χ^2^	*P-*value
	High-High* (n = 22)	High-Low (n = 94)	Low-High (n = 199)	Low-Low (n = 634)	Other** (n = 1733)		
**Demographical**							
**Age (years)**							
Median (IQR)	35 (21–47)^a^	45 (32–56)^b^	44 (30–57)^b^	48 (34–57)^c^	41 (30–53)^b^	F = 185.38	<0.001^#^
Sex, n (%)							
Male	985 (69.60)^a^	270 (71.42)	229 (76.84)	215 (63.23)	1,001 (63.27)^b^	χ^2^ = 33.10	<0.001
Female	431 (30.40)^a^	108 (28.58)	69 (23.16)	125 (36.77)	581 (36.73)^b^		
**Occupation, n (%)**							
Farmer	1,136 (80.20)^a^	265 (70.10)^a^	212 (71.14)^a^	247 (72.64)^a^	1,080 (68.27)^b^	χ^2^ = 57.79	<0.001
Non-farmer	280 (19.80)^a^	113 (29.90)^a^	86 (28.86)^a^	93 (27.36)^a^	502 (31.73)^b^		
**Ecological and Socioeconomic**							
Mean Elevation (m)	576.01^ab^ (451.17–700.25)	250.94^a^ (190.66–311.21)	675.15^b^ (577.41–772.90)	207.49^a^ (177–45–237.52)	1,020.36^c^ (963.39–1077.34)	F = 82.50	<0.001
Mean monthly precipitation (mm)	106.82^a^ (97.45–116.19)	101.40^a^ (95.26–107.55)	120.67^b^ (117.40–123.93)	76.88^c^ (74.57–79.19)	62.79^d^ (61.08–64.49)	F = 167.25	<0.001
Rural-type counties^†^ (%)	100.00	93.61 (86.45–97.11)	91.45 (86.66–94.63)	76.02 (72.54–79.19)	86.60 (84.9–88.1)	χ^2^ = 58.43	<0.001
Mean pig density (head/km^2^)	212.20^a^ (146.40–278.00)	212.49^a^ (181.57–243.41)	134.28^b^ (114.48–154.09)	190.50^a^ (176.43–204.58)	88.68^b^ (83.25–94.11)	F = 78.40	<0.001
Mean cattle density (head/km^2^)	7.88^a^ (4.14–11.62)	24.18^ab^ (18.06–30.29)	23.54^ab^ (19.73–27.35)	36.36^b^ (31.26–41.46)	19.62^ab^ (17.55–21.70)	F = 14.41	<0.001
Mean farmland production (kg/ha)	2,949.67^ab^ (1953.41–3945.93)	3,372.04^b^ (2854.01–3890.06)	1,457.58^c^ (1267.94–1647.22)	4,296.41^c^ (4080.49–4512.33)	2,315.05^a^ (2208.14–2421.97)	F = 99.84	<0.001
GDP^‡^	440.80^a^ (236.61–644.98)	3,070.73^b^ (2042.65–4098.83)	1,427.95^c^ (1025.29–1830.60)	4,448.88^d^ (4006.23–4891.54)	1,974.07^c^ (1787.85–2160.30)	F = 41.99	<0.001

Note: *High-High (High-risk counties): a county identified if only as HH based on LISA for more than 50% of the period of study. **Other: not statistically significant cluster as determined by LISA.

Results expressed as mean (95% CI) unless otherwise noted;

^#^Kruskal-Wallis test.

^†^Type of each county (rural or urban) was defined based on the predominant proportion of area. The proportion of area was calculated from mean values of pixels of raster maps of each county polygon^51^.

^‡^Unit: RMB 10,000 (Chinese Yuan).

^a,b,c,d^Different letter denotes significant difference after post hoc Tukey’s HSD adjustment between value between clusters at level ≤0.05.

IQR, interquartile range; GDP, gross domestic product.

Elevation, precipitation, type of county, livestock density, farmland production and gross domestic product (GDP) was significantly differed between clusters (p < 0.001). The high-risk clusters were situated in areas at higher elevation (576.01 meter; 95% CI: 451.17–700.25, p < 0.001) and higher precipitation rate (136.86 mm per month; 95% CI: 123.61–150.12, p < 0.001) compared to low-risk clusters. High-risk clusters were more rural (100%) than the other type of clusters (p < 0.001). Pig density was not differed among high-risk (212.20 head/km^2^, 95%CI: 146.40–278.00) and low-risk clusters (190.50, 95%CI: 176.43–204.58), but it was still higher than the other clusters. Cattle density in high-risk clusters was much lower (7.88 head/km^2^, 95%CI: 4.14–11.62) than that low-risk clusters (36.36 head/km^2^, p < 0.001). Both receptive clusters (high-low and low-high clusters) had moderate livestock density. The high-risk clusters had lower farmland production (2,949.67 kg/ha; 95% CI: 1,953.41–3,945.93 p < 0.001) compared to low-risk clusters (4,148.50 kg/ha 95% CI: 3,951.64–4,345.36). Additionally, the GDP of high-risk clusters was much lower (440.80 Yuan, 95% CI: 236.61–644.98, p < 0.001) than that in low-risk clusters (4,448.88 Yuan, 95% CI: 1,025.29–1,830.60).

## Discussion

We analyzed notified human leptospirosis data from 2005 to 2016 in China to determine the spatiotemporal geographical distribution in incidence rates, to identify residual high-risk counties for leptospirosis and most importantly to profile the demographical, ecological and socioeconomic characteristics between high-risk and low-risk counties. Overall, although there was a gradual decline in the notified leptospirosis incidence and a reduction in the number of counties reporting leptospirosis during the period studied, our analysis has revealed residual counties with high leptospirosis incidence in the southwestern, central and southeastern China. Additionally, our study demonstrates important demographical, ecological and socioeconomic differences between high-risk and low-risk counties which could form the basis of future disease elimination strategies. These findings highlight the need for targeted interventions that account for local determinants to further reduce the burden of leptospirosis in China.

Our analysis reveals persistently high incidence in a limited set of counties in the south Yunnan including Mengla County in Xishuangbanna prefecture and Menglian County in Pu’er prefecture which border with Myanmar and Lao P.D.R (Luang Namtha province). These findings are also have regional significance since leptospirosis is also highly prevalent in Myanmar and Lao P.D.R^29–31^. The high incidence of leptospirosis in this area may be linked to shared climatic and local socio-ecological characteristics. For example, Xishuangbanna prefecture is characterized by tropical and monsoonal climate, which provide favorable conditions for *Leptospira* environmental survival. In addition to paddy fields, approximately 30% of the total land area of Xishuangbanna prefecture is covered by rubber plantations^32^. The majority of the population is involved in cash crops plantations (e.g., rubber, tea, corn, rice) as well as small-scale pig farming^33^. Rural communities in this area are known as the poorest populations with the annual GDP per capita less than US$100. Uncontrolled cross-border live animal trade such as pigs, cattle and buffalo has potential on the spread of some zoonotic diseases including leptospirosis since these species are known to be important reservoir for particular pathogenic *Leptospira* serovars^9,34^. Hence, targeted intervention should be implemented on these high-risk areas and the communities living along the Mekong river basin. Transboundary disease monitoring programs both in humans and livestock animals should be prioritized to control leptospirosis, especially in the border between Yunnan, Lao P.D.R, and Myanmar. Further research will be carried out to better understand key factors that drive leptospirosis transmission in these high-risk counties at local-level.

Despite a remarkable decrease in leptospirosis rates in the last decade^16,17^, our analyses demonstrated significant annual spatial clustering of leptospirosis cases. Yet, our annual estimates of clustering (as measured by Moran’*I* statistics) indicate a significant reduction in the tendency for leptospirosis clustering with time. This may partly be explained by considerable control efforts as well as ecological and social changes that occurred during the last few decades in China^35^ which bring endemic areas to a lower endemicity level and *on par* with low endemicity areas surrounding them. Substantial preventive and control actions have been promoted including rodent control programs and vaccination especially in endemic areas^22,23^. Also, significant investment to improve hygiene and sanitation infrastructure^36,37^ throughout the country might also have helped at reducing the geographical extent of leptospirosis risk in China.

The observed changes in the geographical distribution of leptospirosis risk could be also linked with landscape changes that have been undergone in China^38^. Of note, over the past three decades, China experienced a large-scale modification in landscape due to industrialization and urbanization^39–41^, which may have impacted directly or indirectly the spatial distribution of leptospirosis. China’s land cover has substantially impacted by national-scale reforestation policy known as Grain for Green Program^42^ which to some extent this might have changed vegetation structure and the diversity and population dynamics of host animals including rodents, leading to changes in the distribution of leptospirosis risk. In addition, ecological impact due to the development of Three Gorges Dam might have also altered rodent abundance^43^ and this might reduce the transmission risks in that affected areas. It was evidenced by low level incidence in Hubei and Chongqing in this study, which also in agreement with existing local study^44^. Moreover, a recent seroprevalence survey in the Three Gorges Dam region has also indicated that *Leptospira* prevalence in host animals especially in rodents was low^45^. The geographical changes in leptospirosis risk could be also due to changes in human behaviors. In China’s rural areas, where leptospirosis is endemic, modernization had triggered substantial changes in farming practices via mechanization. This change might have reduced the level of exposure to leptospiral contaminated water or soil. Further local investigation is essentially required in the high-risk counties identified in this study to assess the impact of landscape and social changes on the spatial variation of risk of leptospirosis.

Our analysis identified persistent spatiotemporal clusters of local leptospirosis in China during 2005 to 2016. Most of the high-risk counties were spatially clustered in the tropical and sub-tropical region in south China comprises 12 provinces such as Guangdong, Guangxi, Zhejiang, Anhui, Fujian, Jiangxi, Hubei, Hunan, Chongqing, Sichuan, Yunnan, and Guizhou. Those provinces situated along China’s major river basin such as Yangtze, Lancang (upper Mekong) river and Pearl river. Based on our findings, the persistent leptospirosis hotspots that exist over time in southwestern, central and southeastern counties highly suggesting that most leptospirosis incidence in these high-risk areas could be primarily driven by the interplay between agricultural activities, low socioeconomic conditions, rodent proliferation and climate. Our study indicates that in high-risk counties, leptospirosis was observed in younger population and greater proportion in males and farmers compared to low-risk counties; suggesting that intervention in the residual high-risk counties should be more focused on this active population group that engage with agricultural activities. Our findings also indicated that high-risk counties had ecological and socioeconomic characteristics that also common in areas where leptospirosis is endemic. High-risk counties were economically less-developed and were more rural situated in moderate elevation with higher precipitation compared to low-risk counties. Interestingly, livestock population density and farmland production in high-risk counties was much lower than that of low-risk areas which suggest that family or subsistence small holder farming system may play an important role in human infection in that high-risk counties; however, the role of rodents and livestock animals as important source of infection cannot be discarded and it deserves further local investigations. To illustrate, in Guizhou, it was identified that *L. interrogans* serogroup Icterohaemorrhagiae serovar Lai was predominantly identified in rodent *A. agrarius*^18^. In Pan’an county in Zhejiang, *Rattus confucianus* and *R. flavipectus* were found to be dominant and potential source of leptospiral infection^46^. In addition, several major outbreaks in high-risk counties identified in this study following heavy rainfall leading to flooding have been reported, including in Sichuan^24^ and Anhui^47^, highlighting the importance of rainfall and flooding on leptospirosis risk.

While the evidence presented in this study can be beneficial to help identify areas where surveillance and interventions should be directed, there are some study limitations that need to be considered. We incorporated all cases (i.e., suspect, clinically diagnosed and laboratory confirmed leptospirosis cases) in our analyses to allow comparison with Chinese government reports and local studies. However, as this study used leptospirosis notification data collected from a passive surveillance system, it has the potential to greatly underestimate the actual incidence rates as our dataset merely captures individuals who seek medical treatment. There could be a number of individuals who represent subclinical, mild influenza-like symptom and did not aware and/or unable to look for treatment immediately, especially in remote and poor rural areas in China. In addition, there might also variation in awareness and diagnostic capacity among physicians and hospitals over time and space, which could misrepresent the spatial extent of the disease.

In summary, our study reveals for the first time the dynamic pattern of leptospirosis distribution in China and identified a small set of persistent high-risk counties in China indicating an opportunity for success of leptospirosis interventions towards elimination in China. Intervention strategies should be more targeted to communities living in less developed rural areas, particularly in that high-risk counties identified in this study.

## Materials and Methods

### Ethics statement

The study was approved by the Medical Research Ethics Committee of the University of Queensland (#2016001608) and the Ethics Committee of Beijing Institute of Disease Control and Prevention. All records were anonymized and aggregated to county-level prior to the commencement of analysis. The de-identification method was performed in accordance with the relevant guidelines and regulations for the de-identification of protected health information. No personal identifiers were present and maps presented in this paper do not identify patients’ addresses. Signing of a consent form was not necessary as secondary data were used and the participants were not identified.

### Data collection and management

#### Infection Data

We utilized notified human leptospirosis data that has been used in our previous study elsewhere^16^. Briefly, in China, leptospirosis has been classified as Class B Notifiable Disease since 1955. All diagnosed cases of leptospirosis must be reported by all healthcare providers at county-level to the Center for Disease Control and Prevention through the China Information System for Diseases Control and Prevention (CISDCP). Notified leptospirosis cases include information about sex, age, occupation, date of onset of illness, date of diagnosis, date of death, case classification (suspected, clinical, and laboratory-confirmed), and address. Leptospirosis cases are defined into three categories: suspected, clinical, and confirmed case^48^. Suspected cases are defined as an individual with: a) one of the following clinical symptoms such as acute fever (up to 39 °C) which may be accompanied by chills, myalgia, or malaise and; b) history of exposure within a month prior to the onset of illness to the following risk factors: epidemic season, reside in epidemic area, either direct or indirectly contacted with suspected animals and their urine or feces or contaminated water and soil. Clinical (probable) cases are defined as suspected cases with at least one of the following clinical manifestations: conjunctival hyperemia, gastrocnemius tenderness, or enlargement of the lymph nodes. A confirmed case is defined as a suspected case with one or more any of the following laboratory criteria: 1) positive culture of *Leptospira* from blood, urine, tissues, or cerebrospinal fluid (CSF); 2) microscopic agglutination test (MAT) titre of ≥400 in single or paired serum samples; 3) a fourfold or greater rise in MAT titers between acute and convalescent-phase samples; 4) presence of pathogenic *Leptospira* spp detected by polymerase chain reaction (PCR); 5) presence of IgM antibodies by enzyme-linked immunosorbent assay (ELISA). All cases reported from 1^st^ January 2005-31^st^ December 2016 were included in our analyses.

For the purpose of spatial analyses, all individual leptospirosis cases were linked to respective county-level polygons based on county code using the geographical information systems (GIS) software (ArcGIS version 10.5.1, ESRI Inc., Redlands, CA, USA). The mainland China comprises 31 provinces/autonomous region/municipalities and more than 2,900 counties, with population size ranging from 7,123 to 5,044,430 people and geographic area size ranging from 5.4 to 197,346 square kilometers.

#### Ecological and socio-economic characteristics data

Leptospirosis risk is perceived to be multifactorial in nature involving complex interactions between ecological and socio-economic conditions^10,12^. Elevation data and monthly precipitation data with 30 arc-seconds (~1-km) spatial resolution was extracted from WorldClim (v.2) (available at www.worldclim.org), which was based on the average meteorological data for 1970–2000^49,50^. An urban extent grid (v.1) raster dataset was obtained from the Global Rural-Urban Mapping Project (GRUMP v.1)^51^ and used to determine the proportion of urbanized or rural areas of each county (http://sedac.ciesin.columbia.edu/data/set/grump-v1-urban-extents). Data for pig and cattle density for each county was sampled from Gridded Livestock of the World version 2.01 with 1-km spatial resolution retrieved from FAO-GeoNetwork (http://www.fao.org/geonetwork/srv/en/main.home)^52^. Farmland productivity raster map were obtained from the Resource and Environmental Science Data Center of the Chinese Academy of Sciences (http://www.resdc.cn)^53^. Socioeconomic condition of each county was indicated by the gross domestic product (GDP). A raster map of 2010 Gross Domestic Product (GDP) of China with 1-km resolution was used (http://www.geodoi.ac.cn/weben/doi.aspx?Id=125)^54^. Zonal mean values for each raster datasets were sampled at each county polygon using Zonal Statistics module in the Spatial Analyst toolbox in ArcGIS software.

### Data analyses

#### Descriptive analysis and disease mapping

A county-level notified human leptospirosis cases were analyzed descriptively and overall yearly notified leptospirosis and number of county reported leptospirosis were plotted. Number of leptospirosis cases of each county was then utilized to explore the spatial distribution of the leptospirosis in China. A county-level crude standardized morbidity ratio (SMR) was estimated by dividing the observed number of cases by the expected number of cases in the study population (overall incidence rate of human leptospirosis for the whole country from 2005 to 2016 multiplied by the population of each county)^55^. County-level population data for 2005–2016 were obtained from the National Bureau of Statistics of China. To reduce random variation resulting from a small number of observations and to produce statistically more precise risk estimates, spatial smoothing based on empirical Bayes method was applied (defined as smoothed SMRs), so that the effect of different population sizes in corresponding county can be adjusted^56,57^. The empirical Bayes smoothing procedure was implemented using *R* software package ‘*DCluster’*.

#### Global and local spatial autocorrelation statistics

To determine the presence of spatial dependence in the smoothed SMRs across counties during the period studied, global Moran’s *I* statistics was calculated. As proposed by Assunção and Reis^58,59^, Moran’s *I* statistics were adjusted based on the Empirical Bayes Index. Moran’s *I* value ranging from −1 to 1 with a value close to 0 indicates no spatial clustering (random). A positive value indicates positive autocorrelation and a negative value means negative autocorrelation^60^. A spatial weight matrix was constructed based on *k*-nearest neighbors approach^59^. The significance of Moran’s *I* of smoothed rates was assessed using Monte-Carlo randomization with 999 permutations. Significance (p < 0.05) of the test statistic indicates that incidence is spatially clustered or dispersed. Moran's I calculation was performed under R environment on package ‘spdep’^61,62^.

Local indicators of spatial association (LISA) analysis was performed as the global pattern was not random. LISA was calculated to detect the presence of clusters of counties with high (High-high, HH) and low rates (Low-Low, LL), as well as spatial outliers (High-Low, HL and Low-High, LH). HH clusters are defined when a county with a high value of leptospirosis incidence is surrounded by other counties also with high values leptospirosis incidence (later classified as high-risk county)^63^. While LL clusters represent counties with low values of leptospirosis incidence surrounded by neighboring counties with low values of leptospirosis incidence (classified as low-risk county). The High-Low or Low-High clusters indicates counties with high or low incidence surrounded by counties with low or high incidence. From a spatial epidemiology point of view, the spatial outliers can explain whether the area defined as receptive area (Low-High) or endemic area (High-Low). Low-High areas are expected to be vulnerable to disease introduction as they are surrounded by high-risk areas. In contrast, High-Low areas may play an important role in spreading the disease to their low-risk neighbors and the probability of transmission is a function of both share similar underlying epidemiological conditions that may favor infection spread. LISA analysis was carried out by using GeoDA ver. 1.8 software^64^.

Maps were created using ArcGIS v10.5 (ESRI, Redlands, CA, USA).

### Statistical analysis

Descriptive analyses were performed to profile and compare demographical, ecological, and socioeconomic conditions of all cluster categories (e.g., High-high, HH; Low-Low, LL; Low-High, LH; High-Low, HL; and Other, insignificant cluster) as identified by LISA analysis during 12-year period studied. Continuous variables (e.g., age, elevation, precipitation, pig density, cattle density, farmland production and GDP) were described using their mean and 95% confidence interval (CI) or median and interquartile range (IQR). Categorical variables (e.g., sex, occupation type, type of county) were described as count and proportions and 95%CI. Differences in case demographic information, ecological and socioeconomic conditions between clusters were tested either using χ^2^ tests (for categorical variables) or one-way ANOVA or Kruskal-Wallis test with post hoc Tukey’s honestly significant difference (HSD) test (for continuous variables). Levels of significance were set at 5%. All statistical analyses were performed using SPSS 24 (IBM Corp, Armonk, NY, USA).

## Electronic supplementary material

Supplementary Table A

## Data Availability

The datasets that support the findings of this study are available from China CDC but restrictions apply to the availability of these data, which were used under license for the current study, and so are not publicly available. Interested parties can apply for the data by contacting the data center of China public health science or email data@chinacic.cn. The metadata generated or analysed during this study are included in this published article (and its Supplementary Information file).
