# Supplementary material for: Geographical and temporal distribution of the residual clusters of human leptospirosis in China, 2005–2016
Source: Sci Rep. 2018 Nov 9;8:16650. doi: 10.1038/s41598-018-35074-3 (PMC6226456; doi:10.1038/s41598-018-35074-3)
Supplement: Supplementary file 1 — Supplementary Table A [file 41598_2018_35074_MOESM1_ESM.docx]

**Geographical and temporal distribution of the residual clusters of human leptospirosis in China, 2005-2016**

Pandji Wibawa Dhewantara^1,2^, Abdullah Al Mamun^3^, Wen-Yi Zhang^4^, Wen-Wu Yin^5^, Fan Ding^5^, Danhuai Guo^6^, Wenbiao Hu^7^, Ricardo J. Soares Magalhães^1,8^

^1^ UQ Spatial Epidemiology Laboratory, School of Veterinary Science, The University of Queensland, Gatton, QLD 4343, Australia;

^2^ National Institute of Health Research and Development (NIHRD), Ministry of Health of Indonesia, Pangandaran Unit of Health Research and Development, West Java 46396, Indonesia

^3^ Institute for Social Science Research, The University of Queensland, Indooroopilly, QLD 4068, Australia.

^4^ Center for Disease Surveillance and Research, Institute of Disease Control and Prevention of PLA, Beijing 100071, People’s Republic of China.

^5^ Chinese Center for Disease Control and Prevention, Beijing 102206, People’s Republic of China.

^6^ Scientific Data Center, Computer Network Information Center, Chinese Academy of Sciences, Beijing 100190, People’s Republic of China.

^7^ School of Public Health and Social Work, Queensland University of Technology, Kelvin Grove, QLD 4059, Australia

^8^ Children’s Health and Environment Program, Child Health Research Centre, The University of Queensland, South Brisbane, QLD 4101, Australia

**Corresponding authors:**

**Pandji Wibawa Dhewantara,** UQ Spatial Epidemiology Laboratory, School of Veterinary Science, The University of Queensland, Gatton, QLD 4343, Australia. Tel: +61 467930433. Email: p.dhewantara@uq.edu.au;

**Wen-Wu Yin,** Division of Infectious Diseases, Chinese Center for Disease Control and Prevention, 155 Changbai Road, Changping District, Beijing 102206, China.

Email: yinww@chinacdc.cn

**Supplementary Table A**. Yearly notified human leptospirosis cases, proportion of laboratory confirmed cases and number of county reported, 2005-2016

| Year | No. of cases (n=8158) | Incidence rate (1/100000) | % confirmed cases | No. of county reported |
| --- | --- | --- | --- | --- |
| 2005 | 1465 | 0.11 | 8.2 | 307 |
| 2006 | 717 | 0.05 | 31.7 | 265 |
| 2007 | 958 | 0.07 | 27.9 | 299 |
| 2008 | 929 | 0.07 | 26.8 | 278 |
| 2009 | 659 | 0.05 | 24 | 222 |
| 2010 | 718 | 0.05 | 31.9 | 239 |
| 2011 | 423 | 0.03 | 42.1 | 182 |
| 2012 | 491 | 0.04 | 37.4 | 180 |
| 2013 | 436 | 0.03 | 54.3 | 165 |
| 2014 | 556 | 0.04 | 57.7 | 173 |
| 2015 | 411 | 0.03 | 56.7 | 163 |
| 2016 | 395 | 0.03 | 58.2 | 171 |
